# Maternal perceptions of breastfeeding and infant nutrition among a select group of Maasai women

**DOI:** 10.1186/s12884-018-2165-7

**Published:** 2019-01-07

**Authors:** Marie Dietrich Leurer, Pammla Petrucka, Manjale Msafiri

**Affiliations:** 1 College of Nursing (Regina), University of Saskatchewan, #100 – 4400 4th Avenue, Regina, Saskatchewan S4T 0H8 Canada; 2 College of Nursing, University of Saskatchewan, Regina, Canada; 3 Masters Student Green Hope Organization, Arusha, Tanzania

**Keywords:** Breastfeeding, Infant nutrition, Maasai, Tanzania

## Abstract

**Background:**

Adverse health outcomes are higher among Maasai children in the Ngorongoro Conservation Area compared to other co-located ethnic groups and regions of Tanzania. The Mama Kwanza Socioeconomic Health Initiative, a Canadian-Tanzanian partnership delivering healthcare at clinics in this region, gathered perceptions of mothers regarding breastfeeding and infant nutrition in order to inform culturally sensitive, realistic, and effective health promotion efforts.

**Methods:**

A qualitative description approach was used in interviewing 30 Maasai mothers of infants zero to six months of age to explore their infant feeding practices, beliefs, knowledge, and recommendations to support breastfeeding. A local research team was trained to conduct and transcribe the interviews and assist with data interpretation. Qualitative content analysis was used in analyzing the interview transcripts.

**Results:**

Lactation is universal in this culture with all the mothers planning to breastfeed for at least one year and most having initiated breastfeeding within one hour of birth. Lactation skills and knowledge are passed down intergenerationally from the elder women. None of the infants less than six months were exclusively breastfed, with a variety of liquid and semi-solid supplements given. Mothers perceived their milk alone was nutritionally insufficient with maternal dietary deficiencies cited as a factor.

**Conclusions:**

While there is a strong breastfeeding culture among the Maasai in Ngorongoro, intersectoral efforts are required to provide culturally respectful health education on the benefits of exclusive breastfeeding and to ensure the maternal dietary adequacy required to achieve this goal. The findings reinforce the importance of international health projects adapting health promotion initiatives to local realities and beliefs in efforts to improve maternal child health.

**Electronic supplementary material:**

The online version of this article (10.1186/s12884-018-2165-7) contains supplementary material, which is available to authorized users.

## Background

Health outcomes in Tanzania vary across gender, geographic, and ethnic lines with Maasai pastoralists in northern Tanzania being particularly disadvantaged [1]. Within this context, the Mama Kwanza Socio-Economic Health Initiative (MKSHI), a Tanzanian-Canadian partnership, operates health clinics in the Ngorongoro Conservation Area (NCA) of northwest Tanzania. The NCA has a population of approximately 50,000 people, the vast majority of whom are Maasai pastoralists [2]. Despite an influx of tourists, the poverty rates among the NCA Maasai population remain high with an estimated 58% of households in NCA classified as poor, very poor, or destitute [2]. Factors behind the high poverty rates include drought, livestock disease, fluctuations in market prices, and policies such as a ban on cultivation and restrictions on pastoral practices [2, 3].

The overall infant mortality rate in the Tanzania northern zone encompassing Arusha and NCA was 38 per 1000 for the ten years preceding the 2015–2016 national survey [4]. Research assessing child health among the Maasai in the nearby Arusha and Manyara areas of Tanzania found Maasai children were substantially more vulnerable and reportedly experience diarrhea, pneumonia, and fever more frequently when compared to other co-located ethnic groups [1]. Maasai children were also two to three times more likely to exhibit stunted growth and wasting compared to the other ethnicities, with 80% of Maasai households classified as severely food insecure [1]. The Maasai children also had higher rates of stunting (57% versus 45%) and wasting (10% versus 5%) compared to the national average during the same time period [1, 5]. In 2008, the leading causes of death for children under-five at a rural hospital serving primarily Maasai people in the Ngorongoro District were pneumonia, malaria, diarrheal diseases, neonatal conditions, and malnourishment [6].

One of the most effective strategies to reduce morbidity and mortality in children under-five is promotion and implementation of the World Health Organization’s (WHO) recommendations for infant and young child feeding which include: (a) initiation of breastfeeding within one hour of birth; (b) exclusive breastfeeding (EBF) for the first six months of life; and (c) introduction of safe and nutritionally adequate solid foods at 6 months with continued breastfeeding until at least two years of age [7]. A meta-analysis found delayed breastfeeding initiation (more than an hour after birth) contributed to twice the infant mortality risk in the first month of life compared to infants breastfed within the hour [8]. EBF, defined as “no other food or drink, not even water, except breast milk” although infants can receive vitamins, minerals, oral rehydration salts and medicines [9], enhances the positive benefits accrued through breastfeeding. A systematic review found an increased all-cause mortality risk for non-breastfed (RR 14.4), partially breastfed (RR 4.8), and predominantly breastfed (RR 1.5) compared to EBF infants [10]. Non-EBF and inadequate complementary feeding contribute to increased infant morbidity and mortality from infectious diseases and increased risk of stunting, a factor in poor motor and cognitive development, with greater risk reduction corresponding to more intense breastfeeding practices [11–13].

The evidence suggests increased adoption of WHO infant feeding recommendations could reduce infant morbidity and mortality rates in the NCA. However, several factors found to influence Maasai nutritional decision-making, including the level of poverty/food insecurity, cultural beliefs and traditions, and gender relations, must be assessed when planning health promotion efforts [14]. Food insecurity has been reported among Maasai women in the NCA [15], a factor that can adversely affect maternal willingness to EBF [16]. Research into Maasai cultural practices in other regions found prolonged breastfeeding was the norm, however infants were commonly fed ghee (butter), animal’s milk, herbs and blood prior to six months of age, inconsistent with EBF recommendations [17, 18]. And finally, complex intra- and extra-household gender dynamics and decision-making were found to impact food supply decisions in the polygamous Maasai culture [14].

While clinic staff at the MKSHI clinic anecdotally report low rates of EBF, there has been no known research exploring the breastfeeding and infant nutrition practices among the Maasai in NCA, and more critically, their perceptions, beliefs, and recommendations. Such knowledge will provide guidance for the development of culturally sensitive, realistic, and effective health promotion efforts at the MKSHI clinics. To gather maternal perceptions, qualitative interviews were conducted with mothers of infants attending one of the MKSHI clinics in the Olbalbal district of NCA. The research objectives were to determine: (1) Typical breastfeeding initiation and duration timeframes; (2) Sources of breastfeeding information and awareness of WHO infant nutrition recommendations; (3) Maternal attitudes towards breastfeeding and the WHO recommendations; (4) Timing and nature of introductory liquid and solid foods; and (5) Maternal recommendations to assist breastfeeding mothers.

## Methods

Individual qualitative interviews were conducted with Maasai mothers using a qualitative description approach as described by Sandelowski [19, 20]. Qualitative description is a naturalistic form of inquiry that produces findings closely grounded in the data with a lower level of interpretation [19, 20]. This approach was felt to be appropriate since high level interpretation would be difficult due to the possible loss of meaning through translation and the use of a novice, local Maasai research team member for data collection.

The MKSHI clinic in the Olbalbal district of NCA was chosen as the study site due to the more traditional lifestyle practices of the Maasai people in this unique and remote service location compared to the Maasai population served by the project’s clinic in Arusha. On average, 11 to 12 Maasai received health services daily at this small, rural clinic. Purposive sampling, which focuses on relatively small samples, selected purposefully for their knowledge and experience of the topic of interest, was used in this research [20–22]. Participants were recruited via two purposive sampling strategies, as a combination of strategies is considered appropriate for qualitative research that seeks to apply the results to practice [22]. First, maximum variation criterion sampling, commonly used in qualitative description research, was used with all mothers of infants zero to six months of age attending the clinic during the recruitment period invited to participate thereby maximizing diversity of experiences, maternal age ranges, and parities while seeking commonalities across participants to determine typical infant nutrition practices [20, 22]. Second, snowball sampling occurred as mothers shared the recruitment handout with other eligible mothers who then came to the clinic to volunteer [21, 22]. The use of snowball recruitment made it difficult to estimate non-participation rates since it is unknown how many eligible women may have been informally shown a handout but declined to participate.

The interview guide (Additional file 1) included a mix of open-ended and closed-ended questions exploring infant feeding patterns and the beliefs/rationale behind these, knowledge of current WHO infant nutrition recommendations, current/desired breastfeeding support, and demographic characteristics. Open-ended questions elicited information regarding beliefs, context, and rationale for reported practices. Some examples include: Why did you decide to breastfeed? Who helped you learn about breastfeeding? Is there anything that would help mothers’ breastfeed longer? Closed ended questions explored the details of infant feeding practices. For example: Does your baby eat any solid food? (if no) At what age will you start giving your baby solid food? What is the first solid food you will give your baby? (if yes) How old was your baby when you started giving him/her solid food? What was the first solid food you gave your baby?

A three-person local Maasai research team was hired and trained by the lead author to facilitate data collection and interpretation sensitive to local cultural and linguistic traditions. The lead author, a member of the MKSHI project team who provides strategic advice on health promotion initiatives, has previous experience on qualitative research projects involving interviews with mothers. The receptionist at the MKSHI clinic in Olbalbal served as the recruiter by distributing and describing the recruitment handout to potential participants. A well-known local Maasai woman conducted the audiotaped 20 to 30 min, one-time interviews in Maa in a private room of the clinic. A community member with English writing and computer skills translated the interviews from Maa language into written English transcripts.

Several ethical considerations were addressed in the conduct of this research. First, consent for this study was obtained by the second author from local community leaders. Second, a phone credit voucher worth 5000 TSH (approximately 2.20 USD), an amount consistent with other Tanzanian research among vulnerable populations [23], was provided as an incentive in this minimal risk research. While such an incentive in a poverty context may be an inducement, it has been argued this is not coercive and does not compromise the validity of the consent if the participant has the capacity to make a competent and rational evaluation of the risks and benefits of research participation [24–26]. The researchers in this study do not believe the incentives impacted the candor with which mothers answered the interview questions. Third, potential participants were assured their decision of whether to participate would not affect the services they receive at the clinic. Finally, as per the consent procedure approved by the ethics board, individual participant consent was obtained with the interviewer reading the consent form to participants in Maa due to high rates of illiteracy. Participants who were unable to sign their name wrote an “X” and the interviewer wrote the participant name beside it before signing and dating the consent form. The consent form included the researchers’ goals in conducting the research as well as the benefits and risks to participants.

Initial data analysis was performed by the first author and was reviewed by the second author, with data analysis beginning after the first few interviews had been conducted to ensure the interview guide was collecting the information necessary to meet the research objectives. The researchers acknowledge that their status as nurses working for an academic institution in a western country, and being from a different cultural background, could influence their interpretations. Thus, local MKSHI project team members assisted in interpreting the meaning of the narratives, particularly related to unique cultural practices and attitudes.

Qualitative content analysis (QCA) was used since it is “the analysis strategy of choice in qualitative descriptive studies” [20]. It is a dynamic analysis form that summarizes the data, with the findings remaining closer to the data and less transformed than the level of interpretation typical of more in-depth forms of qualitative data collection [19, 20]. Similar answers to each interview question were organized into data derived groupings based on the holistic meaning of the narratives. As patterns became apparent, these quotation groupings were divided into sub-categories. Interpretations of the issues that arose across more than one category emerged as themes.

## Results

Thirty interviews were conducted in February and March 2015 with recruitment ceasing once analysis revealed saturation with replication evident (most participants providing similar responses across the interviews) [27]. The age of participants ranged from 17 to 40 years with a median age of 26 years old. Infant ages ranged from one to six months (average 4.4; median 5). Participant birth locations were reported as 53% home birth and 47% health clinic birth. Parity was 23% primiparous and 77% multiparous (see Table 1). Since all of the deliveries were at home or at the local clinic, which is not equipped for major surgical interventions, it can be concluded that all the deliveries were vaginal.

**Table 1:** Participant demographic characteristics

Infant	
Age in months	Number of infants
1	2
2	3
3	4
4	3
5	8
6	10
Maternal	
Age in years	Number of participants
17–19	4
20–22	5
23–25	3
26–28	12
29–31	2
32–34	2
35+	2
Parity	Number of participants
1st child	7
2nd child	1
3rd child	9
4th child	6
5th child	4
6th child	2
7th child	1

The participants provided details of their infant feeding practices, including the timing of breastfeeding initiation and planned cessation, supplemental liquids provided, and current or anticipated first solid foods. They also described their rationale for these behaviours and their recommendations for what would help to support mothers in their breastfeeding efforts.

### Breastfeeding practices

#### Timing of breastfeeding initiation

Women were asked how soon after birth they began breastfeeding. The majority of women met the WHO recommendations to begin breastfeeding within an hour of delivery. Only four women initiated breastfeeding more than one hour after birth, with the delay attributed to feeling unwell post childbirth.

*“After delivery I stayed four hours before breastfeeding the baby because I felt dizziness for three hours.”*

*“I did not breastfeed for some days as I was sick and unconscious.”*

Although three of the four instances of delayed initiation occurred subsequent to a home birth, the vast majority of participants initiated breastfeeding within an hour regardless of whether childbirth occurred at home or in a health clinic. Prelacteal feeding was reported in one instance, with a home birth mother describing how she “gave butter first and then breastfed”.

#### Breastfeeding duration

Mothers were asked how long women in their community typically breastfeed their infants. Extended breastfeeding was normal with participants responding that mothers universally breastfeed beyond the first year, and frequently beyond the second year. Over half of the mothers reported that extended breastfeeding for at least three years was not uncommon.

### Breastfeeding knowledge and beliefs

#### Reason for breastfeeding

All thirty mothers were breastfeeding their infants at the time of the interview. Mothers were asked why they had decided to breastfeed. The most common rationales were that it was recommended by the elder women and that it was healthy nutrition for the baby.

*“I was advised by the elder women, but again, it’s a strong way of upbringing babies.”*

*“Because breastfeeding is the only food for newly born.”*

*“I know that breastfeeding is good food for babies”*

#### Source of breastfeeding information

The guidance of the elder women was evident when participants were asked who had helped them learn to breastfeed. While some reported that healthcare professionals provided advice, the most common answer was that breastfeeding knowledge was passed down intergenerationally.

*“First I learned from elder women together with my grandmother”*

*“My mother-in-law.”*

*“The doctors, but I already knew from observing other women”*

#### Knowledge and perception of exclusive breastfeeding recommendations

To discern knowledge of current infant feeding guidelines, mothers were asked if they knew EBF was recommended for the first six months, and what their reaction was to this recommendation. The majority of mothers reported they were not aware of the EBF recommendation. While a few stated the recommendation was a good idea, many felt this was not realistic in their circumstances and expressed the belief that mother’s milk alone was never adequate to provide for an infant’s needs. Other’s suggested the inadequacy of their milk was directly linked to the poor maternal nutrition they experienced.

*“Yes, I know. I think it is a good idea since mother’s milk is better than other kinds of milk like cows.”*

*“Yes, I know but we need to give other liquid food because we are not well fed. We miss balanced food as our cattle also starve. We provide cow’s milk and butter to our babies because breastfeeding alone is not enough. We are afraid of hunger for our children.”*

*“I didn’t know. It is not good as mother’s milk alone is not enough for the baby to fully grow.”*

#### Recommendations to support breastfeeding

To guide program planning at the MKSHI Clinic, mothers were asked what could be done to better support mothers in their breastfeeding efforts. One mother alluded to gender roles in articulating that “good care should be provided by the husband”. Most responded that a healthy and sufficient maternal diet was the most critical factor in supporting EBF, and that this was lacking.

*“Balanced diet and taking heed over the doctor’s advice.”*

*“Meat, fat, soup from goat and sheep.”*

*“Only balanced diet will help.”*

### Supplementation and complementary foods

#### Supplemental liquids given

Mothers were asked if their baby was receiving anything other than mother’s milk. None of the twenty infants under six months of age were EBF since all were receiving a liquid (including semi-solids) in addition to breastmilk. Butter (semi-solid) and goat/cow’s milk were the most common supplements provided to the infants at the time of the interview. Honey, juice, and water were also given with most infants receiving more than one supplemental liquid.

*“My baby receives milk, honey and butter.”*

*“I give milk and butter”*

*“Milk and juice”*

#### Introduction of solid foods

To discern compliance with infant feeding recommendations related to the introduction of complementary solid foods at six months of age, mothers were asked “At what age will/did you start giving your baby solid food?” Few of the six month old infants were receiving any solid food. Most mothers of infants five months and younger intended to delay introduction of solids until eight to nine months of age. It was common to introduce more than one food once they began complementary feeding of solids, with ugali (maize porridge) identified as the most common first solid food.

*“At 8 months old I will start giving beans, meat and other soup.”*

*“When he has teeth, like when he is 9 months old, ugali and beans.”*

## Discussion

The findings provide insight into the infant feeding behaviours of Maasai women in this region. They also highlight the beliefs and rationale behind these behaviours while describing what participants perceive might help mothers to adopt the recommended EBF. Three themes emerged that provide guidance for health promotion programming.

### Breastfeeding is normalized

All 30 mothers were breastfeeding and most anticipated continuing for two years and beyond, illustrating that breastfeeding appears to be universal for women in this community. This is similar to research from other regions that found extended breastfeeding for at least two years is common among the Maasai [18]. Globally, many women experience difficulties with breastfeeding techniques, such as poor latch and sore nipples, leading to early lactation cessation [28, 29]. The participants of this study did not mention such problems, suggestive of a strong breastfeeding support system. Most participants reported the elder women had urged them to breastfeed and were the primary source of breastfeeding information. This contrasts with many western cultures where intergenerational lactation knowledge was disrupted when wide-spread use of formula feeding was adopted for several generations.

Ideally breastfeeding should be initiated within one hour after birth [7]. This appears to be consistent with Maasai cultural practices as the majority of the women began breastfeeding their infant within this timeframe, despite just over half of the participants having home births. Although one participant reported giving butter as a prelacteal feed, typically only colostrum, then breastmilk, were given during the first week or two. This finding is supported by other research reports that found 98% of Maasai babies received colostrum, a higher rate than some other ethnic groups in Northern Tanzania [1].

### Exclusive breastfeeding is not widely practiced

EBF until six months of age, as recommended by WHO, was not widely practiced as all the infants less than six months of age were receiving supplemental liquids (including semi-solids). Consistent with our findings, other research has found that butter is traditionally given to Maasai infants starting at two weeks of age [30]. A barrier to EBF from birth to 6 months is the participants’ belief that their milk alone is insufficient nutrition for their infant. Participants were clear that improved maternal diet was required to boost the adequacy of their milk given the food insecurity experienced in this region. Similarly, Webb-Girard et al. found food insecure women in Kenya were significantly more likely to believe their milk was insufficient to EBF for the first six months [16].

Participant reports of inadequate maternal diet are supported by other research findings of food insecurity among Maasai women in this region [15]. Cultural and socioeconomic factors impact maternal nutritional status in the NCA. Traditional Maasai dietary practices prohibit the consumption of fish, chicken, and wild animals [30]. Intake of most vegetables is also uncommon as greens are viewed as livestock feed. Meat intake is limited because, although the Maasai are pastoralists, livestock are only slaughtered for special occasions [18, 30]. Research has identified gender roles as another factor behind maternal food insecurity among the Maasai, since men’s nutritional needs take priority, followed by the children, with women’s needs of lesser importance, further worsening maternal food insecurity [15, 30]. Finally, the Maasai tradition of valuing land for grazing rather than crop production may negatively impact food supply, with this view reinforced by government policies banning cultivation in the NCA [2]. This policy, combined with the impact of climate change/drought conditions on livestock pasturing, have resulted in high rates of poverty and food insecurity in the region. Families are increasingly reliant on government food aid, primarily maize, contributing to a lack of nutritional diversity and limited intake of fruits and vegetables [18, 31, 32].

Maternal nutritional status, in turn, affects the composition and volume of human milk. While some nutrient content, such as calcium, is independent of maternal diet, others such as vitamins A and B6 are highly dependent on maternal nutritional status [33]. Research with lactating women in pastoral communities in Kenya found the volume of mother’s milk consumed by infants was related to the mothers’ body composition, and concluded “there is a possibility that lactating mothers practicing EBF living under harsh conditions may experience periods of low breastmilk volume” [34]. Some evidence suggests that provision of supplemental food can increase milk production and the duration of EBF among undernourished women [35].

Increased EBF among the Maasai of NCA could have a positive impact since more intensive breastfeeding is associated with reduced incidence of respiratory and diarrheal infections, leading causes of infant mortality in this region [6, 11]. EBF could also reduce the risks of bacterial and viral infections acquired by infant consumption of raw goat/cow milk, reportedly a normal practice among this group of women [36]. Webb-Girard et al. suggested EBF promotion in food insecure populations would be more effective if accompanied by efforts to improve food security [16]. They acknowledged a need to change educational strategies, given the insensitivity of counselling EBF among women with no means to achieve food security, and called for advocacy to ensure nutritional support for lactating women [16].

### Delayed introduction of complementary food

While mothers in this study typically introduced the first solid foods at eight or nine months of age, the WHO recommends complementary foods be introduced at 6 months of age [7]. The finding that maize porridge was a typical first complementary food for infants in this study is consistent with other reports. Research in rural, central Tanzania assessing infant feeding practices and anthropometric measures found porridge was the prevailing complementary meal with complementary food provision characterized by low nutrient content, small portion size, lack of diversity, and meal infrequency, all known contributory factors in the high prevalence of stunting [37].

The WHO has advised the use of fortified complementary foods and vitamin-mineral supplements for infants and young children, if required, including multiple micronutrient powders containing at least iron, vitamin A, and zinc for home fortification of complementary foods, particularly in households where there is a lack of animal source foods [7]. Bhutta et al. reviewed interventions to promote infant and young child nutrition in populations with insufficient food and identified evidence-based core interventions including food supplements or conditional cash transfers in addition to micronutrient supplements [38].

The Tanzanian Ministry of Health and Social Welfare has set objectives to improve breastfeeding rates and the nutritional status and practices of lactating mothers. The main activities identified to achieve this end include disseminating nutritional guidelines and training healthcare workers on optimal maternal, infant and young child feeding. While activities also include scaling up supplementation of Vitamin A as part of routine immunisation efforts, the root causes of maternal food insecurity and lack of nutritional diversity are not directly addressed [39]. Additional evidence-based policies the government could consider include provision of food supplements and conditional cash transfers to undernourished, lactating women, and distribution of fortified complementary foods and vitamin-mineral supplements to food insecure infants and young children as part of efforts to improve infant and maternal nutritional status. Finally, changes to NCA policy allowing limited cultivation such as small gardens would allow Maasai families to sustainably address nutritional insufficiency and lack of diversity [2].

For the MKSHI, the findings highlight Webb-Girard’s recommendation that EBF promotion amidst maternal food insecurity should be accompanied by efforts to ensure adequate nutrition for lactating women [16]. Given the lack of adoption of WHO’s infant feeding recommendations evident in the findings, the MKSHI should facilitate increased education of local health care workers, parents, traditional healers/ birth attendants, and the broader community regarding the health benefits of adopting WHO’s nutritional recommendations, although such efforts must be sensitive to cultural traditions [18]. However, education alone is insufficient without attempts to seek sustainable, community-driven solutions to the food insecurity currently experienced by the Maasai community in NCA. It is critical for the MKSHI team to work with community leaders and government officials to explore long term approaches to address the maternal nutritional insufficiency reported by the women.

This study highlights several areas for further research. First, research exploring the perceptions of the elder women regarding infant nutrition practices would provide important information on the contextual and traditional cultural beliefs behind these practices. Second, comparisons of the breastfeeding practices of mothers delivering at home versus the health center could assess for the impact of delivery location. Finally, future research could explore infant feeding practices and beliefs in more depth throughout the NCA and make comparisons with Maasai communities in other regions.

Trustworthiness of qualitative research and QCA analysis considers several aspects. [40–42]. First, credibility of the findings was reinforced by inviting all mothers with infants 0 to 6 months of age attending the clinic during the study period to participate, resulting in a diverse sample comprised of participants with personal knowledge of the research topic. Credibility was enhanced by having a local Maasai research team conduct data collection in a manner that was respectful of the local context [41]. Second, confirmability of findings relates to establishing that the data interpretation is representative of what participants relayed. The local research team were consulted to ensure the two lead researcher’s interpretations of the transcript statements reflected what the local researchers understood was the meaning of transcript statements based on their knowledge of Maasai customs and beliefs [42]. Confirmability was also enhanced by including direct (translated) quotations from the interviews in support of the findings. Finally, transferability of the findings was facilitated by sufficient description of the context to allow readers to decide if the findings might apply to their setting.

A limitation of this study is that although the findings provided sufficient detail regarding current infant feeding practices to inform health promotion efforts, the mothers did not provide expansive answers due to the Maasai interviewer being a novice qualitative interviewer with beginner-level probing skills. Additional data collection methods and/or multiple interviews would have provided more detailed, in-depth information regarding beliefs and attitudes.

## Conclusion

This research has provided valuable local knowledge regarding the infant nutrition practices and beliefs among a select group of women attending one of the MKSHI clinics in the NCA. Their descriptions and insights revealed that, while breastfeeding is universal, there are cultural and socioeconomic barriers adversely impacting the provision of optimal infant nutrition as recommended by the WHO. The findings offer guidance for the MKSHI in its efforts to promote maternal, infant and child health, and reaffirm the importance of developing health promotion strategies informed by the beliefs, realities, and recommendations of local populations and in partnership with the community.

## Additional file

Additional file 1: Interview Guide. The interview guide contains open-ended and closed-ended questions exploring mothers’ infant feeding behaviours with supporting rationale and their recommendations. (DOCX 13 kb)

## Abbreviations

EBF: Exclusive breastfeeding
MKSHI: Mama Kwansa Socio-Economic Health Initiative
NCA: Ngorongoro Conservation Area
QCA: Qualitative Content Analysis
WHO: World Health Organization
