# Supplementary material for: Maternal perceptions of breastfeeding and infant nutrition among a select group of Maasai women
Source: BMC Pregnancy Childbirth. 2019 Jan 7;19:8. doi: 10.1186/s12884-018-2165-7 (PMC6323693; doi:10.1186/s12884-018-2165-7)
Supplement: Supplementary file 1 — Interview Guide. The interview guide contains open-ended and closed-ended questions exploring mothers’ infant feeding behaviours with supporting rationale and their recommendations. (DOCX 13 kb) [file 12884_2018_2165_MOESM1_ESM.docx]

**Interview Guide**

***Exploring Breastfeeding Perspectives among Women in Northern Tanzania***

- What age is your baby?
- Is this your first baby? IF NO - How many other babies have you had?
- What is your age?
- Where was your baby born?
- Have you ever breastfed this baby?

**IF NO:**

- Why didn’t you breastfeed? Did anyone else help you make this decision?
- What does your baby drink?
- Does your baby eat any solid food?
  - IF NO
    - At what age will you start giving your baby solid food?
    - What is the first solid food you will give your baby?
  - IF YES
    - What solid food do you give your baby?
    - How old was your baby when you started giving him/her solid food?
- Did you know the doctors and nurses recommend mothers give their babies only breastmilk for the first six months?
- What do you think of that?

**IF YES:**

- How soon after birth did you first breastfeed your baby?
- Why did you decide to breastfeed?
- Are you still breastfeeding? If yes, does your baby receive any other liquid?
- Who helped you to learn about breastfeeding?
- Does your baby eat any solid food?
  - IF NO
    - At what age will you start giving your baby solid food?
    - What is the first solid food you will give your baby?
  - IF YES
    - What solid food do you give your baby?
    - How old was your baby when you started giving him/her solid food?
- Did you know the doctors and nurses recommend mothers give their babies only breastmilk for the first six months?
- What do you think of that?
- How long do most women in your community breastfeed their babies?
- Is there anything that would help mother’s breastfeed longer?
